# Supplementary material for: Drosophila activins adapt gut size to food intake and promote regenerative growth
Source: Nat Commun. 2024 Jan 4;15:273. doi: 10.1038/s41467-023-44553-9 (PMC10767106; doi:10.1038/s41467-023-44553-9)
Supplement: Supplementary file 1 — Supplementary Information [file 41467_2023_44553_MOESM1_ESM.pdf]

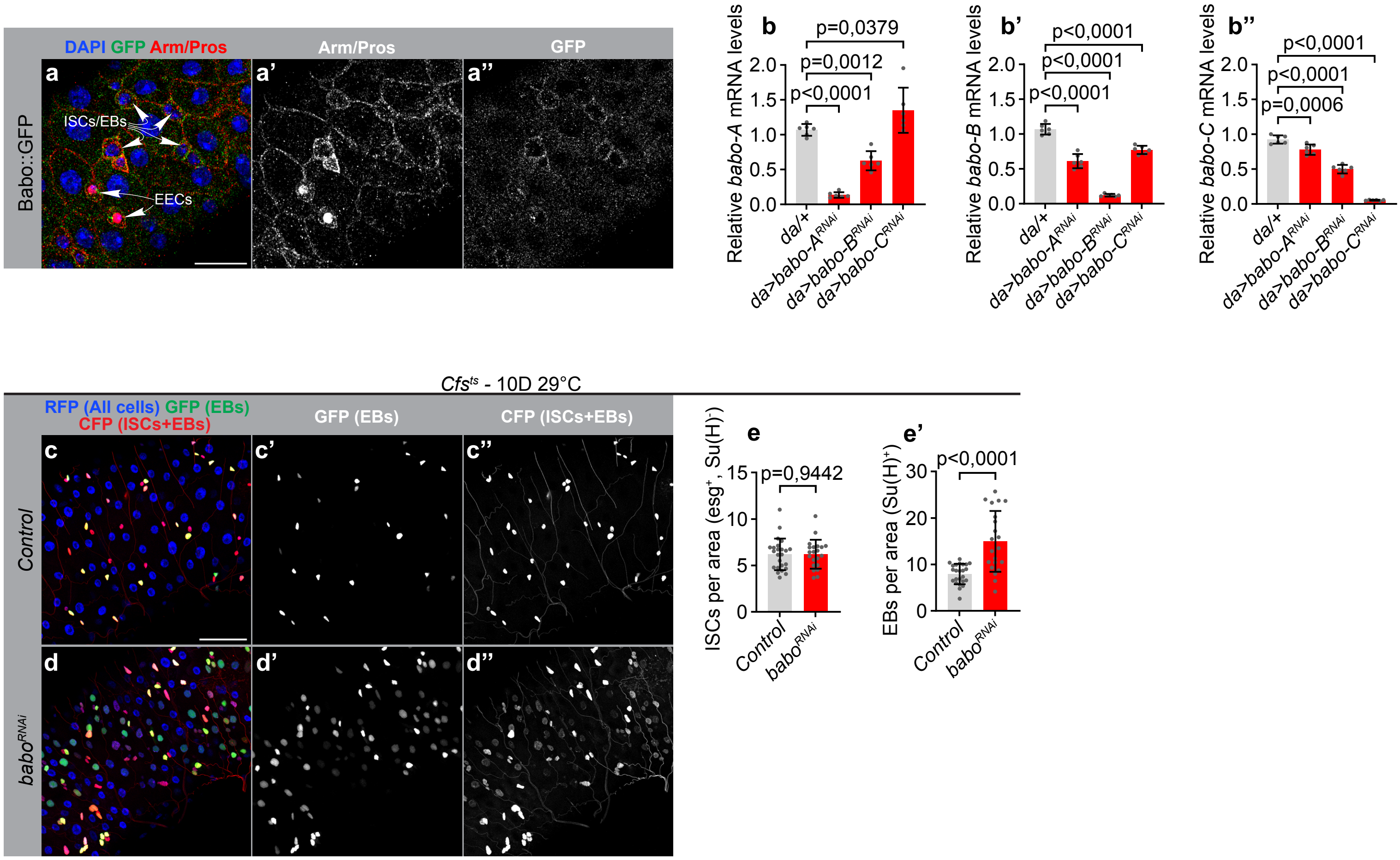

DAPI GFP Arm/Pros
Arm/Pros
GFP
Babo::GFP
a
a'
a''
ISCs/EBs
EECs
b
b'
b''
Relative babo-A mRNA levels
Relative babo-B mRNA levels
Relative babo-C mRNA levels
p=0,0379
p=0,0012
p<0,0001
p<0,0001
p<0,0001
p<0,0001
p<0,0001
p<0,0001
p=0,0006
2.0
1.5
1.0
0.5
0.0
da/+
da>babo-A^RNAi
da>babo-B^RNAi
da>babo-C^RNAi
Cfs^ts - 10D 29°C
RFP (All cells) GFP (EBs)
CFP (ISCs+EBs)
GFP (EBs)
CFP (ISCs+EBs)
c
c'
c''
d
d'
d''
Control
babo^RNAi
e
e'
ISCs per area (esg+, Su(H)-)
EBs per area (Su(H)+)
p=0,9442
p<0,0001
15
10
5
0
30
20
10
0
Control
babo^RNAi

Supplementary Figure 1

(a-a'') The Type I Activin receptor *babo* is expressed in the midgut. (a) Confocal image of a posterior midgut from flies carrying GFP-tagged Babo (Babo::GFP) under the control of its endogenous promoter stained for GFP (a, a''), Armadillo (Arm) and Prospero (Pros) (a, a') and DNA (blue, a) reveal enriched expression in ISCs/EBs and other Pros-positive diploid cells (EECs) (a). (b-b'') RT-qPCR analysis on dissected control midguts with ubiquitous knockdown of isoforms A (b), B (b') and C (b'') demonstrating the specificity of the isoform-specific RNAi lines (n= 6, 6, 6, 6). (c-e') Knockdown of all three Babo isoforms in stem and progenitor cells results in an accumulation of EBs. Quantification of cell type numbers in dissected posterior midguts from control flies and flies with ISC/EB-specific knockdown of all three Babo isoforms using Cfs^{ts} (n= 24, 21). Significance was tested with two-tailed unpaired t-tests (e, e') and one-way ANOVA (b, b', b'') with post-hoc multiple comparison analysis. Data are presented as mean values +/- SD. Source data are provided as a Source Data file.

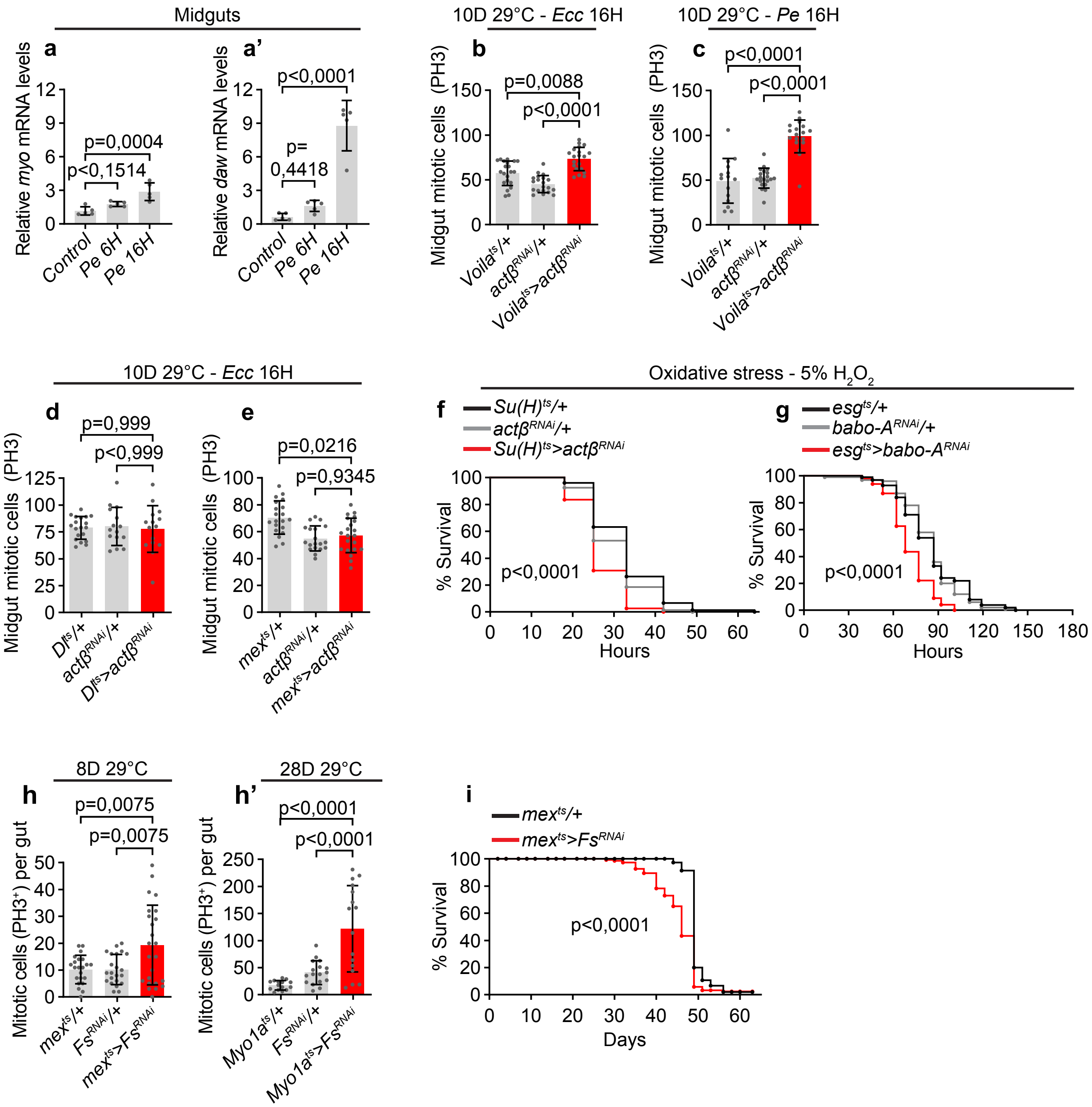
Midguts
a
Relative myo mRNA levels
p=0,0004
p<0,1514
Control
Pe 6H
Pe 16H
a'
Relative daw mRNA levels
p<0,0001
p=
0,4418
10D 29°C - Ecc 16H
b
Midgut mitotic cells (PH3)
p=0,0088
p<0,0001
Voilats/+
actβRNAi/+
Voilats>actβRNAi
10D 29°C - Pe 16H
c
p<0,0001
p<0,0001
10D 29°C - Ecc 16H
d
p=0,999
p<0,999
Dlts/+
Dlts>actβRNAi
e
p=0,0216
p=0,9345
mexts/+
mexts>actβRNAi
Oxidative stress - 5% H2O2
f
Su(H)ts/+
Su(H)ts>actβRNAi
% Survival
p<0,0001
Hours
g
esgts/+
babo-ARNAi/+
esgts>babo-ARNAi
8D 29°C
h
Mitotic cells (PH3+) per gut
p=0,0075
p=0,0075
FsRNAi/+
mexts>FsRNAi
28D 29°C
h'
p<0,0001
p<0,0001
Myo1ats/+
Myo1ats>FsRNAi
i
Days

Supplementary Figure 2

(a-a’) RT-qPCR analysis on dissected control midguts 6- and 16 hours post oral *Pe* infection showing little or moderate upregulation of *Myo* and *Daw* expression, respectively (n= 5, 5, 5). Quantification of mitotic cells ($PH3^+$) in entire midguts dissected from control flies and flies with Actβ knockdown EECs (b-c) (n= 21, 20, 21 and n= 15, 20, 18), ISCs (d) (n= 19, 15, 15) or ECs (d) (n= 20, 19, 21) 16 hours post *Ecc15* (b, d-e) or Pe (c) infection. (f-g) Mated flies with depletion of Actβ in EBs using $Su(H)^{ts}$ (f) (n= 152, 145, 146) or Babo-A in stem and progenitor cells using esg^{ts} (g) (n= 100, 100, 99) display reduced survival to oxidative stress triggered by ingestion of H_2O_2. (h-h’) Quantification of mitotic cells ($PH3^+$) in entire midguts in control flies and flies expressing Fs^{RNAi} in ECs using mex^{ts} or myo^{ts} after 8 (h) (n= 22, 22, 24) or 28 (h’) (n= 15, 17, 16) days of RNAi induction. (i) EC-specific depletion of Fs (Red) shortens lifespan relative to control flies (Black, mated flies were used) (n= 168, 163). Significance was tested with one-way ANOVA (a, a’, c, d, e, h, h’) or Kruskal-Wallis (b) with post-hoc multiple comparison analysis and Mantel-cox Log-rank tests (f, g, i). Data are presented as mean values +/- SD. Source data are provided as a Source Data file.

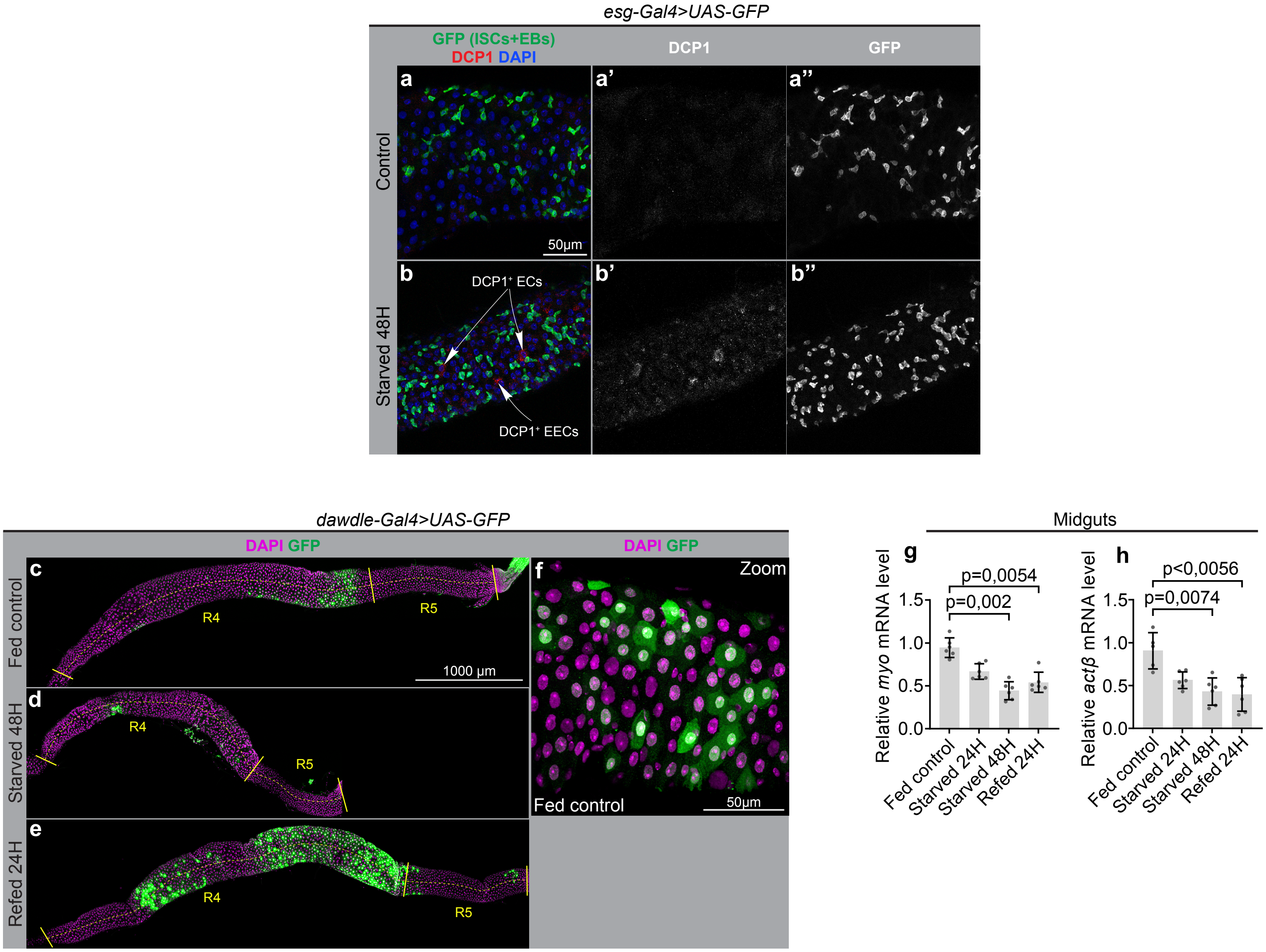
esg-Gal4>UAS-GFP
GFP (ISCs+EBs)
DCP1 DAPI
DCP1
GFP
a
a'
a''
Control
50µm
b
b'
b''
Starved 48H
DCP1+ ECs
DCP1+ EECs
dawdle-Gal4>UAS-GFP
DAPI GFP
c
Fed control
R4
R5
1000 µm
d
Starved 48H
R4
R5
e
Refed 24H
R4
R5
DAPI GFP
f
Zoom
Fed control
50µm
Midguts
g
Relative myo mRNA level
1.5
1.0
0.5
0.0
p=0,0054
p=0,002
Fed control
Starved 24H
Starved 48H
Refed 24H
h
Relative actβ mRNA level
1.5
1.0
0.5
0.0
p<0,0056
p=0,0074
Fed control
Starved 24H
Starved 48H
Refed 24H

Supplementary Figure 3

(a-b'') Representative image of dissected midguts of control (a-a'') or 48H starved (b-b'') animals expressing GFP (in green) in ISCs/EBs and stained for the cleaved effector caspase Dcp-1 (cDcp1, in red) to visualize apoptotic cells. Apoptosis is observed in ECs (large cells), but not in ISCs/EBs (GFP^+ cells). (c-f) Dynamic expression of Daw in ECs in response to starvation and refeeding. Representative confocal image of dissected posterior midgut from control (c), starved (d), and refed (e) flies bearing *daw*-Gal4>UAS-GFP stained for GFP (Green) and DNA (Purple). GFP expression is observed in large polyploidy ECs (f) and is reduced in starved flies and re-induced upon refeeding. (g-h) RT-qPCR analysis monitoring the expression of *myo* and *act*β in midguts dissected from fed, 24H starved, 48H starved, or 48H starved + 24H refed flies (n= 6, 6, 6, 6). Significance was tested with one-way ANOVA (g) or Kruskal-Wallis (h) with post-hoc multiple comparison analysis. Data are presented as mean values +/- SD. Source data are provided as a Source Data file.

Supplemenatry Table 1
Genotypes

Figure 1

a-c	*w; babo-Gal4/+;UAS-GFP/+*
d	*w; esg-Gal4,UAS-GFP/+;TubGal80ts,UAS-Cas9.P2/+*
e	*w; esg-Gal4,UAS-GFP/babo.gRNA;TubGal80ts,UAS-Cas9.P2/+*
f	*w; esg-Gal4,UAS-GFP/+;TubGal80ts,UAS-Cas9.P2/+*
	w; esg-Gal4,UAS-GFP/babo.gRNA;TubGal80ts,UAS-Cas9.P2/+
g	*w; esg-Gal4,UAS-GFP/+;TubGal80ts,UAS-Cas9.P2/+*
	w; esg-Gal4,UAS-GFP/smox.gRNA;TubGal80ts,UAS-Cas9.P2/+
i	*w; esg-Gal4,UAS-mCD8::GFP/+;TubGal80ts,UAS-H2B::RFP/+*
j	*w; esg-Gal4,UAS-mCD8::GFP/+;TubGal80ts,UAS-H2B::RFP/UAS-babo-RNAi*^{29533}
k	*w; esg-Gal4,UAS-mCD8::GFP/UAS-smox-RNAi;TubGal80ts,UAS-H2B::RFP/+*
m	*w; esg-Gal4,UAS-mCD8::GFP/+;TubGal80ts,UAS-H2B::RFP/+*
n	*w/UAS-smox*^{SDVD}*; esg-Gal4,UAS-mCD8::GFP/+;TubGal80ts,UAS-H2B::RFP/+*

Figure 2

a	*w;+;+*
b	*w; esg-Gal4,UAS-mCD8::GFP/+;TubGal80ts,UAS-H2B-RFP/+*
c	*w; esg-Gal4,UAS-mCD8::GFP/UAS-babo-A*^{RNAi}*;TubGal80ts,UAS-H2B::RFP/+*
d	*w; esg-Gal4,UAS-mCD8::GFP/UAS-babo-C*^{RNAi}*;TubGal80ts,UAS-H2B::RFP/+*
h	*w; esg-Gal4,UAS-his2b::CFP,GBE-Su(H)-nlsGFP/ + ;Ubi-his2av::mRFP/ TubGal80ts*
i	*w; esg-Gal4,UAS-his2b::CFP,GBE-Su(H)-nlsGFP/UAS-babo-A*^{RNAi}*;Ubi-his2av::mRFP/ TubGal80ts*
j	*w; esg-Gal4,UAS-his2b::CFP,GBE-Su(H)-nlsGFP/UAS-babo-C*^{RNAi}*;Ubi-his2av::mRFP/ TubGal80ts*
k	*w;Su(H)-Gal4,UAS-GFP/+;TubGal80*^{ts}*/+*
l	*w;Su(H)-Gal4,UAS-GFP/UAS-babo-A*^{RNAi}*;TubGal80*^{ts}*/+*
m	*w;Su(H)-Gal4,UAS-GFP/UAS-babo-C*^{RNAi}*;TubGal80*^{ts}*/+*
o-o’	*w; esg-Gal4,UAS-his2b::CFP,GBE-Su(H)-nlsGFP/+;Ubi-his2av::mRFP/Su(H)-Gal80, TubGal80ts*
p-p’	*w; esg-Gal4,UAS-his2b::CFP,GBE-Su(H)-nlsGFP/ UAS-babo-A*^{RNAi}*;Ubi-his2av::mRFP/Su(H)-Gal80, TubGal80ts*

Figure 3

a	*w; esg-Gal4,UAS-GFP/+;TubGal80*^{ts}*/+*
	w; +; UAS-babo$^{RNAi\ 106092}$*/+*

	w; esg-Gal4,UAS-GFP/+;TubGal80^{ts}/UAS-babo$^{RNAi\ 106092}$
b	*w; esg-Gal4,UAS-GFP/+;TubGal80^{ts},UAS-Cas9.P2/+*
	w; esg-Gal4,UAS-GFP/babo.gRNA;TubGal80^{ts},UAS-Cas9.P2/+
c	*w; esg-Gal4,UAS-GFP/+;TubGal80^{ts}/+*
	w; esg-Gal4,UAS-GFP/UAS-babo$^{RNAi\ 106092}$;TubGal80^{ts}/+
d	*w; esg-Gal4,UAS-GFP/+;TubGal80^{ts}/+*
	w;UAS-babo-A^{RNAi}/+;+
	w; esg-Gal4,UAS-GFP/UAS-babo-A^{RNAi};TubGal80^{ts}/+
	w;UAS-babo-B^{RNAi}/+;+
	w; esg-Gal4,UAS-GFP/babo-B^{RNAi};TubGal80^{ts}/+
	w;UAS-babo-C^{RNAi}/+;+
	w; esg-Gal4,UAS-GFP/UAS-babo-C^{RNAi};TubGal80^{ts}/+
e	*w; esg-Gal4,UAS-GFP/+;TubGal80^{ts}/+*
	w;UAS-babo-A^{RNAi}/+;+
	w; esg-Gal4,UAS-GFP/UAS-babo-A^{RNAi};TubGal80^{ts}/+
f-f’’	*w; esg-Gal4,UAS-his2b::CFP,GBE-Su(H)-nlsGFP/+;Ubi-his2av::mRFP/+*
g-g’’	*w; esg-Gal4,UAS-his2b::CFP,GBE-Su(H)-nls-GFP/UAS-babo-A^{RNAi};Ubi-his2av::mRFP/+*
i	*w;+;+*
j-m’’’	*w;actβ-Gal4,UAS-GFP;GBE-Su(H)-LacZ/+*
n	*w;Su(H)-Gal4,UAS-GFP/+;TubGal80^{ts}/+*
	w;+; UAS-actβ^{RNAi}/+
	w;Su(H)-Gal4,UAS-GFP/+;TubGal80^{ts}/UAS-actβ^{RNAi}
o	*w; esg-Gal4,UAS-GFP/+;TubGal80^{ts}/+*
	w;+; UAS-actβ^{RNAi}/+
	w; esg-Gal4,UAS-GFP/+;TubGal80^{ts}/UAS-actβ^{RNAi}
p	*w; esg-Gal4,UAS-GFP/+;TubGal80^{ts},UAS-Cas9.P2/+*
	w; esg-Gal4,UAS-GFP/actβ.gRNA;TubGal80^{ts},UAS-Cas9.P2/+
q	*w; esg-Gal4,UAS-GFP/+;TubGal80^{ts}/+*
	w;+; UAS-actβ^{RNAi}/+
	w; esg-Gal4,UAS-GFP/+;TubGal80^{ts}/UAS-actβ^{RNAi}
r	*w; esg-Gal4,UAS-mCD8::GFP/+;TubGal80ts,UAS-H2B::RFP/+*
s	*w; esg-Gal4,UAS-mCD8::GFP/UAS-babo-A^{RNAi};TubGal80ts,UAS-H2B::RFP/+*
t	*w; esg-Gal4,UAS-mCD8::GFP/UAS-babo-C^{RNAi};TubGal80ts,UAS-H2B::RFP/+*

Figure 4

a-b	*w;+;+*
c-c’’’	*w; esg-Gal4,UAS-his2b::CFP,GBE-Su(H)-nlsGFP/+;Ubi-his2av::mRFP/+*
d-e	*w;+;+*
f	*w; esg-Gal4,UAS-his2b::CFP,GBE-Su(H)-nlsGFP/+;Ubi-his2av::mRFP/+*

g	*w; esg-Gal4,UAS-his2b::CFP,GBE-Su(H)-nlsGFP/UAS-babo-C*^{RNAi}*;Ubi-hisav::mRFP/+*
j	*w; esg-Gal4,UAS-mCD8::GFP/+;TubGal80ts,UAS-H2B::RFP/+*
k	*w; esg-Gal4,UAS-mCD8::GFP/UAS-babo-C*^{RNAi}*;TubGal80ts,UAS-H2B::RFP/+*
m	*w;mex-Gal4, esg-LacZ/+;TubGal80*^{ts}*/+*
n	*w;mex-Gal4, esg-LacZ/+;TubGal80*^{ts}*/UAS-daw*^{RNAi}
p	*w;mex-Gal4/+;TubGal80*^{ts}*/+*
	w;+;UAS-daw^{RNAi}*/+*
	w;mex-Gal4/+;TubGal80^{ts}*/UAS-daw*^{RNAi}
q	*w; esg-Gal4,UAS-GFP/+;TubGal80*^{ts}*/+*
	w; esg-Gal4,UAS-GFP/UAS-babo-C^{RNAi}*;TubGal80*^{ts}*/+*
r	*w;mex-Gal4/+;TubGal80*^{ts}*/+*
	w;+;UAS-daw^{RNAi}*/+*
	w;mex-Gal4/+;TubGal80^{ts}*/UAS-daw*^{RNAi}

Supplementary figure 1

a-a''	*w;+; Babo::GFP/+*
c-c''	*w; esg-Gal4,UAS-his2b::CFP,GBE-Su(H)-nlsGFP/+;Ubi-his2av::mRFP/+*
d-d''	*w; esg-Gal4,UAS-his2b::CFP,GBE-Su(H)-nls-GFP/+;Ubi-hisav::mRFP/ UAS-babo*^{RNAi}

Supplementary figure 2

a-a'	*w;+;+*
b	*w;TubGal80*^{ts}*/+;Voila-Gal4/+*
	w;+;UAS-actβ^{RNAi}*/+*
	w;TubGal80^{ts}*/+;Voila-Gal4/UAS-actβ*^{RNAi}
c	*w;TubGal80*^{ts}*/+;Voila-Gal4/+*
	w;+;UAS-actβ^{RNAi}*/+*
	w;TubGal80^{ts}*/+;Voila-Gal4/UAS-actβ*^{RNAi}
d	*w; TubGal80*^{ts}*/+;Dl-Gal4,UAS-GFP/+*
	w;+; UAS-actβ^{RNAi}*/+*
	w; TubGal80^{ts}*/+;Dl-Gal4,UAS-GFP/UAS-actβ*^{RNAi}
e	*w;mex-Gal4/+;TubGal80*^{ts}*/+*
	w;+;UAS-actβ^{RNAi}*/+*
	w;mex-Gal4/+;TubGal80^{ts}*/UAS-actβ*^{RNAi}
f	*w;Su(H)GBE-Gal4,UAS-GFP/+;TubGal80*^{ts}*/+*
	w;+/+; UAS-actβ^{RNAi}*/+*
	w;Su(H)GBE-Gal4,UAS-GFP/+;TubGal80^{ts}*/UAS-actβ*^{RNAi}
g	*w; esg-Gal4,UAS-GFP/+;TubGal80*^{ts}*/+*
	w;UAS-babo-A^{RNAi}*/+;+*
	w; esg-Gal4,UAS-GFP/UAS-babo-A^{RNAi}*;TubGal80*^{ts}*/+*

h *w;mex-Gal4/+;TubGal80*^{ts}*/+*

w;UAS-Fs$^{RNAi\ 57394}$*/+;+*

w;mex-Gal4/UAS-Fs$^{RNAi\ 57394}$*;TubGal80*^{ts}*/+*

h’ *w;Myo1a-Gal4,UAS-GFP/+;TubGal80*^{ts}*/+*

w;+;UAS-Fs$^{RNAi\ 46260}$*/+*

w;Myo1a-Gal4,UAS-GFP/+;TubGal80^{ts}*/UAS-Fs*$^{RNAi\ 46260}$

i *w;mex-Gal4/+;TubGal80*^{ts}*/+*

w;mex-Gal4/+;TubGal80^{ts}*/ UAS-Fs*$^{RNAi\ 46260}$

Supplementary figure 3

a-b’’ *w; esg-Gal4,UAS-GFP/+;TubGal80*^{ts}*/+*

c-f *w;daw-Gal4;UAS-GFP*

g-h *w;+;+*
