# Supplementary material for: Drosophila activins adapt gut size to food intake and promote regenerative growth
Source: Nat Commun. 2024 Jan 4;15:273. doi: 10.1038/s41467-023-44553-9 (PMC10767106; doi:10.1038/s41467-023-44553-9)
Supplement: Supplementary file 3 — Reporting Summary [file 41467_2023_44553_MOESM3_ESM.pdf]

Corresponding author(s): Julien Colombani

Last updated by author(s): 2023-04-12

# Reporting Summary

Nature Portfolio wishes to improve the reproducibility of the work that we publish. This form provides structure for consistency and transparency in reporting. For further information on Nature Portfolio policies, see our Editorial Policies and the Editorial Policy Checklist.

## Statistics

For all statistical analyses, confirm that the following items are present in the figure legend, table legend, main text, or Methods section.

| n/a | Confirmed | |
|---|---|---|
| ☐ | ☒ | The exact sample size ($n$) for each experimental group/condition, given as a discrete number and unit of measurement |
| ☐ | ☒ | A statement on whether measurements were taken from distinct samples or whether the same sample was measured repeatedly |
| ☐ | ☒ | The statistical test(s) used AND whether they are one- or two-sided<br>*Only common tests should be described solely by name; describe more complex techniques in the Methods section.* |
| ☒ | ☐ | A description of all covariates tested |
| ☐ | ☒ | A description of any assumptions or corrections, such as tests of normality and adjustment for multiple comparisons |
| ☐ | ☒ | A full description of the statistical parameters including central tendency (e.g. means) or other basic estimates (e.g. regression coefficient) AND variation (e.g. standard deviation) or associated estimates of uncertainty (e.g. confidence intervals) |
| ☒ | ☐ | For null hypothesis testing, the test statistic (e.g. $F$, $t$, $r$) with confidence intervals, effect sizes, degrees of freedom and $P$ value noted<br>*Give P values as exact values whenever suitable.* |
| ☒ | ☐ | For Bayesian analysis, information on the choice of priors and Markov chain Monte Carlo settings |
| ☒ | ☐ | For hierarchical and complex designs, identification of the appropriate level for tests and full reporting of outcomes |
| ☒ | ☐ | Estimates of effect sizes (e.g. Cohen's $d$, Pearson's $r$), indicating how they were calculated |

*Our web collection on statistics for biologists contains articles on many of the points above.*

## Software and code

Policy information about availability of computer code

| | |
|---|---|
| Data collection | ZeissZenBluev3.1 was used for image aquisition |
| Data analysis | Data was analyzed with FIJI/ImageJ v1.53 and Graphpad Prism v9. |

For manuscripts utilizing custom algorithms or software that are central to the research but not yet described in published literature, software must be made available to editors and reviewers. We strongly encourage code deposition in a community repository (e.g. GitHub). See the Nature Portfolio guidelines for submitting code & software for further information.

## Data

Policy information about availability of data

All manuscripts must include a data availability statement. This statement should provide the following information, where applicable:
- Accession codes, unique identifiers, or web links for publicly available datasets
- A description of any restrictions on data availability
- For clinical datasets or third party data, please ensure that the statement adheres to our policy

All source data needed to evaluate the conclusions are present in the paper and are provided as a Source Data file.

## Human research participants

Policy information about studies involving human research participants and Sex and Gender in Research.

| | |
|---|---|
| Reporting on sex and gender | No human studies were performed |
| Population characteristics | No human studies were performed |
| Recruitment | No human studies were performed |
| Ethics oversight | No human studies were performed |

Note that full information on the approval of the study protocol must also be provided in the manuscript.

# Field-specific reporting

Please select the one below that is the best fit for your research. If you are not sure, read the appropriate sections before making your selection.

☒ Life sciences ☐ Behavioural & social sciences ☐ Ecological, evolutionary & environmental sciences

For a reference copy of the document with all sections, see nature.com/documents/nr-reporting-summary-flat.pdf

# Life sciences study design

All studies must disclose on these points even when the disclosure is negative.

| | |
|---|---|
| Sample size | No statistical methods were used to calculate the sample size. The reasoning behind the number of samples for each experiment is that it is large enough to capture normal variation while maintaining experimental feasibility. |
| Data exclusions | No data was excluded in this study |
| Replication | Experiments have been performed at least twice with successful replication. |
| Randomization | Each experimental cohort (genotype) was a pool of siblings that developed and eclosed at the same time and was raised at the same conditions. For comparisons of flies of the same cohort with different treatments (starvation/refeeding/infection), flies were randomly assigned at the start of the treatment. |
| Blinding | No blinding was done during this study |

# Reporting for specific materials, systems and methods

We require information from authors about some types of materials, experimental systems and methods used in many studies. Here, indicate whether each material, system or method listed is relevant to your study. If you are not sure if a list item applies to your research, read the appropriate section before selecting a response.

### Materials & experimental systems

| n/a | Involved in the study | |
|---|---|---|
| ☐ | ☒ | Antibodies |
| ☒ | ☐ | Eukaryotic cell lines |
| ☒ | ☐ | Palaeontology and archaeology |
| ☐ | ☒ | Animals and other organisms |
| ☒ | ☐ | Clinical data |
| ☒ | ☐ | Dual use research of concern |

### Methods

| n/a | Involved in the study | |
|---|---|---|
| ☒ | ☐ | ChIP-seq |
| ☒ | ☐ | Flow cytometry |
| ☒ | ☐ | MRI-based neuroimaging |

## Antibodies

| | |
|---|---|
| Antibodies used | Rabbit anti-PH3 (Milipore 06-570)<br>Chicken anti-GFP (Abcam 13970)<br>Mouse anti-Beta-Galactosidase (DSHB 40-1A)<br>Mouse anti-Armadillo (DSHB N2-7A1)<br>Mouse anti-Prospero (DSHB MR1A) |

| | |
|---|---|
| | Mouse anti-Delta (DSHB C594.9B)<br>Rabbit anti-Dcp-1 (Cell signaling Asp215)<br><br>Alexa Fluor 488-conjugated goat anti-rabbit (Thermofischer #A-11008)<br>Alexa Fluor 488-conjugated goat anti-chicken (Thermofischer #A-21467)<br>Cy3-conjugated donkey anti-mouse (Jackson ImmonuResearch #715-165-151)<br>Cy5-conjugated donkey anti-chicken (Jackson ImmunoResearch #703-175-155) |
| Validation | Validation of the primary antibodies were done previously as these antibodies are extensively used for IHC and are previously published. |

## Animals and other research organisms

Policy information about studies involving animals; ARRIVE guidelines recommended for reporting animal research, and Sex and Gender in Research

| | |
|---|---|
| Laboratory animals | All experiments examined adult Drosophila melanogaster (6+ days after eclosion). Genotypes used in each experiment/figure are detailed in "Genotypes" attached to the manuscript. |
| Wild animals | The study did not involve wild animals. |
| Reporting on sex | All experiments were conducted on female adult Drosophila. Using females is the field standard due to the larger size of their intestines which eases the extraction and analysis of the organ. |
| Field-collected samples | No Field-collected animals were used in the study |
| Ethics oversight | No ethics approval or oversight is required for studies using Drosophila. |

Note that full information on the approval of the study protocol must also be provided in the manuscript.
